# Autoimmune Comorbidities as Modifiers of Phenotypic Heterogeneity in Facioscapulohumeral Dystrophy

**DOI:** 10.1002/acn3.70529

**Published:** 2026-09-22

**Authors:** Jonathan Pini, Giulia Tammam, Andra Ezaru, Manuela Gambella, Benoît Sanson, Luisa Villa, Michele Cavalli, Mihai‐Bogdan Ioncea, Angela Puma, Sabrina Sacconi

**Affiliations:** ^1^ Peripheral Nervous System and Muscle Department Nice University Hospital, Pasteur 2 Hospital Nice France; ^2^ University Cote D'azur Inserm, CNRS, Institute for Research on Cancer and Aging of Nice (IRCAN) Nice France

**Keywords:** autoimmune diseases, disease modifiers, facioscapulohumeral muscular dystrophy

## Abstract

**Objective:**

Facioscapulohumeral dystrophy type 1 (FSHD1) shows clinical heterogeneity that is only partly explained by D4Z4 repeat unit (RU) size. Although immune and inflammatory mechanisms may contribute to disease variability, the prevalence and clinical impact of autoimmune diseases in FSHD remain unclear. We investigated the spectrum of autoimmune comorbidities in FSHD1 and their relationship with D4Z4 repeat size, clinical phenotype, and disease severity.

**Methods:**

We retrospectively analyzed 299 genetically confirmed FSHD1 patients followed at a national neuromuscular reference center. Clinical severity was assessed using the FSHD score and Comprehensive Clinical Evaluation Form classification. Demographic, genetic, and clinical features were compared according to autoimmune disease status. Multivariable linear regression assessed the independent association between autoimmune disease and FSHD severity, adjusting for age, sex, disease duration, D4Z4 RU number, and clinical phenotype.

**Results:**

Eighty‐two patients (27.4%) had at least one autoimmune disease, totaling 96 autoimmune conditions. Several autoimmune diseases were markedly overrepresented compared with published population estimates. Patients with autoimmune disease had larger D4Z4 repeat arrays (7.18 ± 1.82 vs. 6.53 ± 1.81 RU, *p* = 0.002) but higher FSHD severity scores (7.98 ± 3.65 vs. 6.56 ± 3.51, *p* = 0.003). Autoimmune comorbidities were enriched in patients carrying 7–10 D4Z4 RU. In multivariable analysis, autoimmune disease remained independently associated with greater FSHD severity (β = 2.12, 95% CI 1.47–2.76, *p* < 0.0001).

**Conclusions:**

Autoimmune diseases are frequent in FSHD1 and independently associated with greater severity despite larger D4Z4 repeat arrays, supporting immune‐related mechanisms as potential modifiers of FSHD1 expression.

## 1 Introduction

Facioscapulohumeral muscular dystrophy (FSHD) is one of the most common inherited muscular dystrophies, with an estimated prevalence of 1:8000 to 1:20,000 individuals worldwide [1, 2]. It is characterized by progressive weakness of the facial, scapular, and humeral muscles, with highly variable onset and severity, ranging from asymptomatic carriers to severe childhood‐onset disease [3, 4, 5]. Although progression is usually slow, up to 20% of patients eventually require full‐time wheelchair use or become severely disabled [6]. This clinical heterogeneity complicates diagnosis, prognosis, and management [7].

FSHD1 (OMIM # 158900) accounts for approximately 95% of cases and is caused by contraction of D4Z4 macrosatellite repeats on chromosome 4q35, leading to pathological derepression of *DUX4* [8]. FSHD2 (OMIM # 158901) represents about 5% of cases and results from impaired chromatin repression, most often involving *SMCHD1* and/or other modifiers, which also permits *DUX4* expression [9]. Thus, all forms of FSHD converge on aberrant DUX4 expression. DUX4 activation induces transcriptional dysregulation [10, 11], oxidative stress [12, 13], impaired myogenesis [14], immune activation [15], chronic inflammation [16, 17], and muscle cell toxicity [18].

Disease severity is partly associated with D4Z4 repeat size, with shorter repeat arrays generally linked to earlier onset, faster progression, and greater muscle impairment [4, 19, 20]. However, this genotype–phenotype relationship is incomplete, and substantial variability exists even among relatives carrying identical D4Z4 contractions [21, 22]. This suggests that additional genetic, epigenetic, environmental, and immunological modifiers influence disease expression.

Increasing evidence implicates immune and inflammatory mechanisms in FSHD pathogenesis. Muscle biopsies have shown inflammatory infiltrates, mainly composed of CD8+ T lymphocytes and macrophages [23]. MRI studies have identified muscle edema and inflammatory changes, particularly during active disease phases [24, 25, 26]. Transcriptomic analyses have revealed upregulation of innate immune pathways, interferon signaling, cytokine‐related genes, and inflammatory mediators in FSHD tissues [13, 27, 28]. *DUX4* itself can also activate immune‐related pathways and induce antiviral and inflammatory gene expression [15].

Several reports have described associations between FSHD and autoimmune or systemic inflammatory disorders, including inflammatory myopathies [29] and myasthenia gravis [30]. These observations suggest possible shared pathogenic mechanisms or increased immune susceptibility. However, the prevalence of autoimmune diseases in FSHD remains insufficiently characterized, and it is unclear whether these conditions merely coexist with FSHD or contribute to disease severity through chronic systemic inflammation or immune‐mediated muscle injury.

The relationship between autoimmune diseases and FSHD clinical expression is therefore of particular interest. Chronic immune activation may exacerbate muscle degeneration, influence regenerative capacity, or modify the phenotypic expression associated with DUX4 toxicity [31, 32]. Inflammatory pathways may also interact with epigenetic regulation of the D4Z4 locus, potentially contributing to interindividual variability in severity [33, 34].

In this study, we analyzed a large cohort of genetically confirmed FSHD1 patients to characterize the prevalence and spectrum of autoimmune comorbidities and investigate their association with clinical severity, D4Z4 repeat unit number, and Comprehensive Clinical Evaluation Form classification. We further assessed whether autoimmune diseases independently contribute to FSHD severity using multivariable regression models adjusted for demographic, genetic, and clinical factors.

## 2 Methods

This retrospective study was declared and registered on the hospital data processing registry by the Nice University Hospital data protection officer, and compliant with the reference methodology MR004 of the French data protection authority (CNIL). Patients were informed of the use of their data in the present study.

### 2.1 Patients

We included patients aged 18 and older with a confirmed FSHD1 diagnosis (1–10 D4Z4 repeats on chromosome 4qA) who were referred to the Nice University Hospital's National Neuromuscular Diseases Reference Center between 2017 and 2025, and for whom complete demographic data (age at visit, age at onset, sex, and autoimmune comorbidities), clinical (CCEF and FSHD score), and genetic data (D4Z4 repeat unit number) were available.

Autoimmune diseases were identified by reviewing the patients' medical records. Diagnoses had been confirmed by the relevant specialist. For Sjögren's syndrome, only patients fulfilling the 2016 ACR/EULAR classification criteria (score ≥ 4) with positive anti‐SSA and/or anti‐SSB antibodies were included. For inflammatory myopathies, only cases diagnosed after genetically confirmed FSHD1 and supported by myositis‐specific autoantibodies, characteristic muscle biopsy findings, and response to corticosteroids were considered.

### 2.2 Genetic Analysis

The diagnosis of FSHD was clinically established according to the 2010 or 2024 guidelines for genetic diagnosis of FSHD [35, 36] and confirmed by the presence of 1–10 repeat units in the D4Z4 region and a permissive 4qA allele.

### 2.3 Comprehensive Clinical Evaluation Form (CCEF)

The CCEF (available at http://www.fshd.it/documenti/) is a clinical evaluation form containing four sections [37]: the evaluation form (clinical history, disability, muscle involvement), the FSHD evaluation scale (score range 0–15), the clinical diagnostic form (summarizes clinical features), and phenotypic classification. Category A includes patients with typical facial and scapular girdle muscle weakness and is subdivided based on the severity of facial involvement (A1‐A3). Category B includes patients with weakness limited to scapular girdle (B1) or facial muscles (B2). Category C includes pauci‐symptomatic (C1) or asymptomatic patients (C2) without functional impairment (FSHD score = 0); Category D includes FSHD patients with atypical clinical features, either associated with a typical or incomplete FSHD phenotype (subcategory D1) or occurring in pauci‐symptomatic/asymptomatic individuals or in patients not fulfilling criteria for categories A–C (D2). For patients evaluated multiple times, the most recent CCEF was used.

### 2.4 Statistical Analysis

Statistical analysis was performed using GraphPad Prism version 10.6.1 (GraphPad Software, Boston, Massachusetts, USA, www.graphpad.com). Continuous variables were summarized as means ± standard deviations and categorical variables as counts and percentages.

Expected cases were calculated as cohort size × prevalence in the general population. Observed‐to‐expected ratios were calculated by dividing the number of observed cases by the number expected based on published general population prevalence estimates (Table S1). Ninety‐five percent confidence intervals were estimated assuming a Poisson distribution of observed cases.

Continuous variables were analyzed according to autoimmune status in the overall cohort, and within clinical classes or repeat unit classes using unpaired *t*‐tests, with Welch's correction when appropriate. Categorical variables were analyzed using Fisher's exact test. When multiple pairwise comparisons were performed, *p*‐values were adjusted using the false discovery rate (FDR) method according to the Benjamini–Hochberg procedure.

Multivariable linear regression analyses were performed to evaluate the independent association between autoimmune disease and FSHD severity, adjusting for age, sex, disease duration, D4Z4 repeat units, and CCEF categories. Interaction terms were introduced when appropriate to test whether the effect of autoimmune status varied according to D4Z4 repeat units or CCEF categories.

All tests were two‐sided, and *p* < 0.05 was considered statistically significant.

## 3 Results

### 3.1 Demographic and Clinical Characteristics of the Cohort

**FIGURE 1:**
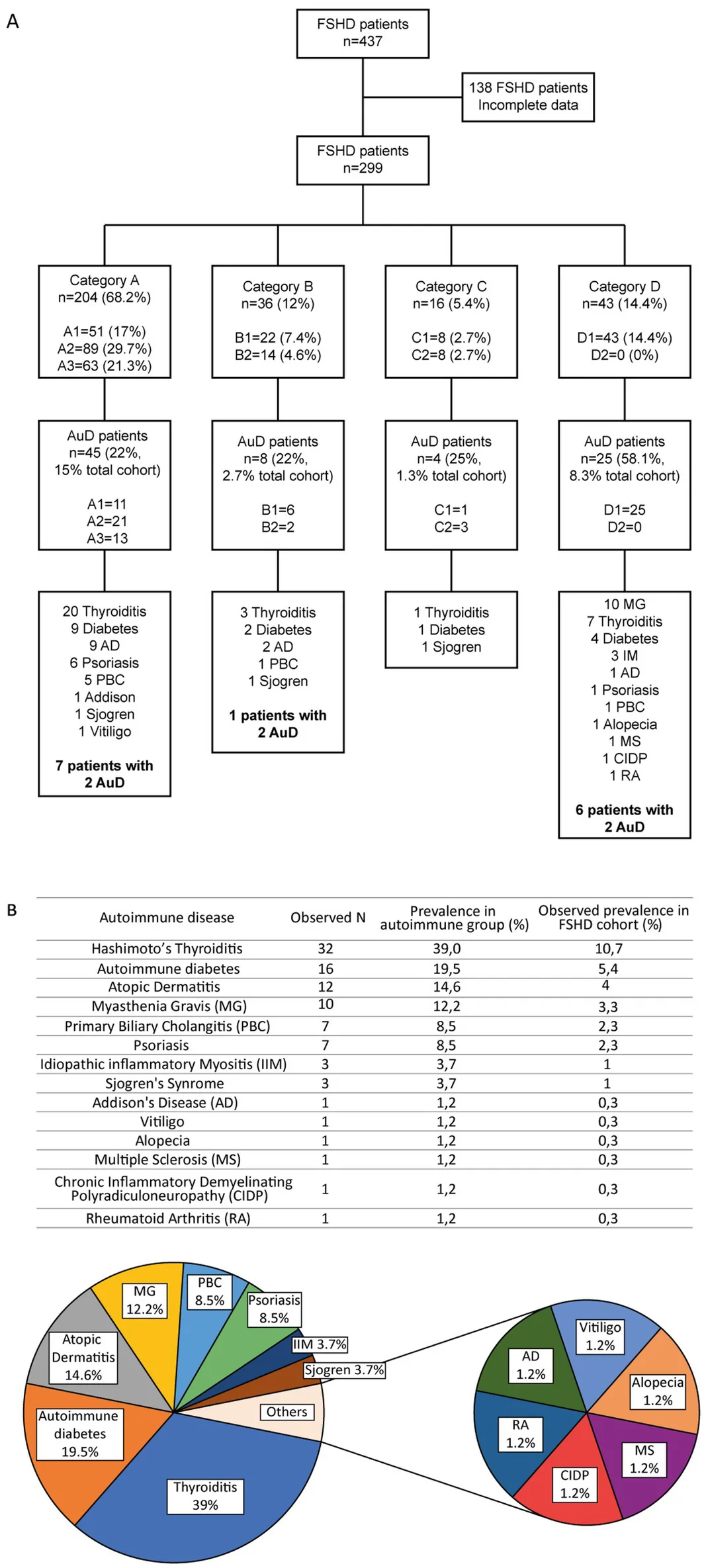
Study design and spectrum of autoimmune diseases in the FSHD1 cohort. (A) Breakdown of patients and distribution of autoimmune diseases across CCEF categories. Patients were classified according to the Comprehensive Clinical Evaluation Form (CCEF) into categories A (typical FSHD phenotype), B (incomplete phenotype), C (asymptomatic/pauci‐symptomatic), and D (atypical phenotype). The number and subtype distribution of autoimmune diseases identified within each category are shown. (B) Distribution of autoimmune diseases identified in the FSHD1 cohort. A total of 96 autoimmune conditions were identified in 82 patients. The table summarizes the number of cases, prevalence among autoimmune patients, and prevalence within the entire FSHD1 cohort. The pie charts illustrate the relative contribution of each autoimmune disease. Hashimoto's thyroiditis was the most common autoimmune comorbidity, followed by autoimmune diabetes, atopic dermatitis, myasthenia gravis (MG), primary biliary cholangitis (PBC), and psoriasis. Less frequent conditions included idiopathic inflammatory myositis (IIM), Sjögren's syndrome, Addison's disease (AD), vitiligo, alopecia, multiple sclerosis (MS), chronic inflammatory demyelinating polyradiculoneuropathy (CIDP), and rheumatoid arthritis (RA).

A total of 437 patients with FSHD1 were initially screened. Among them, 138 patients were excluded due to incomplete datasets, leaving 299 patients with complete data for analysis (Figure 1A). Of these, 167 patients had been previously described [30, 38].

The mean age at examination was 52.6 ± 13.8 years (range: 18–82), and the mean age at onset was 35.0 ± 16.3 years. The cohort was balanced in terms of sex distribution, with 148 males (49.5%) and 151 females (50.5%). The mean D4Z4 repeat unit (RU) number was 6.71 ± 1.83, with the majority of patients carrying 7–10 RUs (56.5%). The mean FSHD clinical score was 6.95 ± 3.6 (Table 1).

**TABLE 1:** Demographic, genetic, and clinical characteristics of the FSHD1 cohort according to autoimmune disease status.

		Total	No autoimmune disease	Autoimmune disease	Statistical significance
	N	299	217	82	
Age (years)	Mean (SD)	52.59 (13.81)	51.22 (14.03)	56.26 (12.55)	*p* = 0.003**
Age at onset (years)	Mean (SD)	34.97 (16.29)	33.7 (15.88)	38.42 (16.99)	*p* = 0.03*
Disease duration (years)	Mean (SD)	17.21 (11.86)	17.1 (11.61)	15.52 (12.58)	NS
Sex	M—N (%)	148 (49.5)	109 (50.23)	39 (47.56)	NS
	F—N (%)	151 (50.5)	108 (49.77)	43 (52.44)	NS
D4Z4 Repeat Unit	Mean (SD)	6.71 (1.83)	6.53 (1.81)	7.18 (1.82)	*p* = 0.002**
	1–3 (%)	15 (5.02)	12 (5.53)	3 (3.66)	NS
	4–6 (%)	115 (38.46)	97 (44.70)	18 (21.95)	*p* = 0.0003***
	7–10 (%)	169 (56.52)	108 (49.77)	61 (74.39)	*p* = 0.0001***
CCEF	A—N (%)	204 (68.23)	159 (73.27)	45 (54.88)	*p* = 0.003**
	B—N (%)	36 (12.04)	28 (12.9)	8 (9.76)	NS
	C—N (%)	16 (5.35)	12 (5.53)	4 (4.88)	NS
	D—N (%)	43 (14.38)	18 (8.29)	25 (30.49)	*p* < 0.0001***
FSHD Score	Mean (SD)	6.95 (3.6)	6.56 (3.51)	7.98 (3.65)	*p* = 0.003**

*Note:* Continuous variables are presented as mean ± standard deviation (SD), and categorical variables as number and percentage. Comparisons between patients with and without autoimmune disease were performed using unpaired *t*‐tests for continuous variables and Fisher's exact test for categorical variables. D4Z4 repeat unit (RU) categories were defined as 1–3, 4–6, and 7–10 repeat units. Clinical severity was assessed using the Comprehensive Clinical Evaluation Form (CCEF), including Category A (classical phenotype), Category B (incomplete phenotype), Category C (asymptomatic/pauci‐symptomatic), and Category D (atypical phenotype). Statistical significance was defined as *p < 0.05, ** *p* < 0.01, ***p < 0.001.

Abbreviation: NS, nonsignificant.

Most patients in our cohort presented the classical FSHD phenotype, characterized by facial and scapular girdle muscle weakness, and were therefore classified as category A (*N* = 204, 68.23%), followed by patients with uncommon features classified as category D (*N* = 43, 14.38%), and patients with muscle weakness limited to scapular girdle or facial muscles, classified as category B (*N* = 36, 12.04%). A minority of our patients were pauci‐symptomatic or asymptomatic, being classified as category C (*N* = 16, 5.35%). Of the 204 patients classified in category A, 51 were classified in subcategory A1, 89 in subcategory A2, and 64 in subcategory A3. In category B, 22 patients were classified in subcategory B1, and 14 patients in subcategory B2. In category C, patients were equally distributed between the two subcategories (8 in each in C1 and C2), while in category D, all patients belonged to subcategory D1 (43). Detailed subclassifications are presented in Figure 1A.

### 3.2 Spectrum and Prevalence of Autoimmune Comorbidities in FSHD1

Among the 299 included patients, 82 (27.4%) had at least one autoimmune disease, including 14 patients with two concomitant autoimmune conditions. Among the 96 recorded autoimmune conditions, 73 (76.04%) occurred in patients carrying 7–10 D4Z4 RU, indicating a predominance of autoimmune comorbidities in patients with larger residual D4Z4 arrays.

According to the CCEF classification, most autoimmune patients were classified in category A (*n* = 45, 15.1% of the total cohort) or category D (*n* = 25, 8.4%), whereas fewer patients were classified as category B (*n* = 8, 2.7%) or category C (*n* = 4, 1.3%; Figure 1A).

A total of 14 distinct autoimmune diseases were identified. Hashimoto's thyroiditis was the most frequent autoimmune condition, affecting 32 patients (39% of autoimmune cases), followed by autoimmune diabetes in 16 patients (19.5%), atopic dermatitis in 12 patients (14.6%), myasthenia gravis in 10 patients (12.2%), and primary biliary cholangitis and psoriasis in 7 patients each (8.5%; Figure 1B).

Comparison with published population prevalence estimates (Table S1) revealed that several autoimmune diseases were overrepresented in the FSHD1 cohort (Table 2). Among the most frequent autoimmune comorbidities, autoimmune diabetes was markedly enriched in FSHD patients, with an observed‐to‐expected (O/E) ratio of 42.81 (95% CI 26.23–69.88). Similarly, myasthenia gravis and primary biliary cholangitis showed strong overrepresentation compared with the general population, with O/E ratios of 167.22 (95% CI 89.97–310.80) and 106.42 (95% CI 50.73–223.22), respectively.

**TABLE 2:** Observed‐to‐expected ratios of autoimmune diseases in the FSHD1 cohort.

Autoimmune disease	Observed N	Observed prevalence in FSHD cohort (*N* = 299)	General population prevalence	Expected N in cohort	O/E ratio	95% CI
Hashimoto's thyroiditis	32	10.7%	7.80%	23.32	1.37	0.97–1.94
Autoimmune diabetes	16	5.40%	0.13%	0.37	42.81	26.23–69.88
Atopic dermatitis	12	4.01%	17.10%	51.13	0.23	0.13–0.41
Myasthenia gravis	10	3.34%	0.02%	0.06	167.22	89.97–310.80
Primary biliary cholangitis	7	2.34%	0.02%	0.07	106.42	50.73–223.22
Psoriasis	7	2.34%	1.92%	5.74	1.22	0.58–2.56
Idiopathic inflammatory myositis	3	1.00%	0.01%	0.04	71.67	23.11–222.21
Sjögren's syndrome	3	1.00%	0.06%	0.18	16.45	5.30–51
Addison's disease	1	0.33%	0.01%	0.03	30.40	4.28–215.85
Vitiligo	1	0.33%	0.20%	0.60	1.67	0.24–11.87
Alopecia	1	0.33%	2%	5.98	0.17	0.02–1.19
Multiple sclerosis	1	0.33%	0.13%	0.40	2.51	0.35–17.85
CIDP	1	0.33%	0.003%	0.01	119.45	16.82–847.98
Rheumatoid arthritis	1	0.33%	0.35%	1.05	0.96	0.13–6.78

*Note:* Observed prevalence corresponds to the proportion of patients affected in the FSHD1 cohort. Expected cases were calculated according to published prevalence estimates from the general population. Observed‐to‐expected (O/E) ratios were calculated by dividing observed cases by expected cases. Ninety‐five percent confidence intervals were estimated assuming a Poisson distribution of observed cases.

Idiopathic inflammatory myositis and Sjögren's syndrome were also enriched in the FSHD cohort, with O/E ratios of 71.67 (95% CI 23.11–222.21) and 16.45 (95% CI 5.30–51.00), respectively. In contrast, common inflammatory skin disorders such as atopic dermatitis and psoriasis were not overrepresented in FSHD patients, with O/E ratios of 0.23 (95% CI 0.13–0.41) and 1.22 (95% CI 0.58–2.56), respectively.

Hashimoto's thyroiditis was the most frequent autoimmune disease identified in the cohort, affecting 10.7% of all FSHD patients and 39% of autoimmune patients, although its prevalence was only modestly increased compared with the general population (O/E ratio 1.37, 95% CI 0.97–1.94).

Due to the very small number of observed cases, estimates for Addison's disease, vitiligo, multiple sclerosis, chronic inflammatory demyelinating polyneuropathy (CIDP), alopecia, and rheumatoid arthritis should be interpreted cautiously despite high observed‐to‐expected ratios for some conditions.

### 3.3 Patients With Autoimmune Comorbidities Are More Severely Affected

**FIGURE 2:**
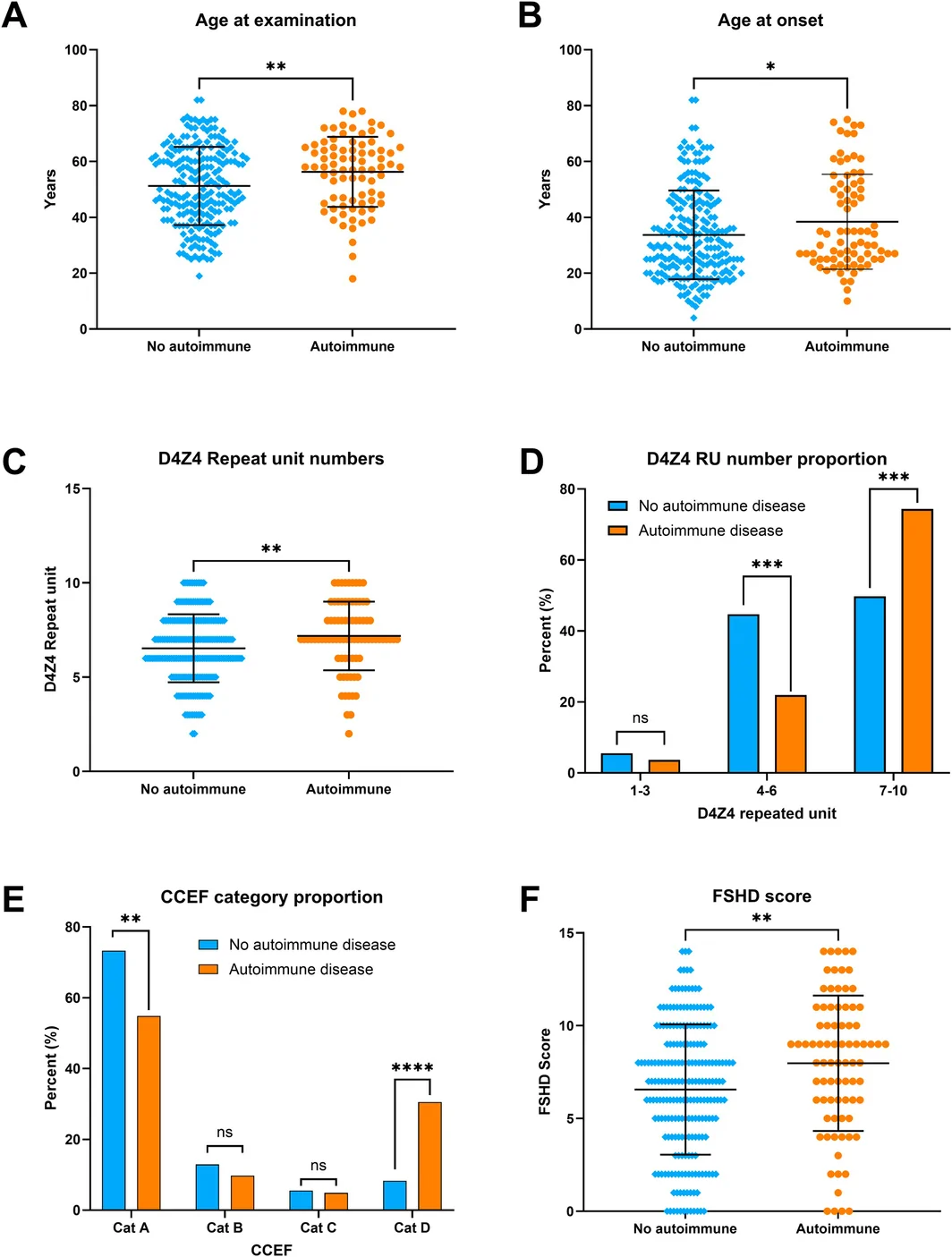
Comparison of demographic, genetic, clinical, and phenotypic characteristics between FSHD1 patients with and without autoimmune disease. (A) Age at examination. (B) Age at disease onset. (C) D4Z4 repeat unit (RU) number. (D) Distribution of D4Z4 RU categories (1–3, 4–6, and 7–10 RU). (E) Distribution of CCEF categories. (F) FSHD clinical severity score. Data are presented as mean ± SD. Statistical significance was assessed using unpaired *t*‐tests or Fisher's exact tests, as appropriate. *p < 0.05, **p < 0.01, ***p < 0.001, ****p < 0.0001.

Patients with autoimmune disease were significantly older at examination compared with patients without autoimmune disease (56.3 ± 12.6 vs. 51.2 ± 14.0 years, *p* = 0.003). Similarly, age at onset was significantly higher in autoimmune patients (38.4 ± 17.0 vs. 33.7 ± 15.9 years, *p* = 0.03), whereas disease duration did not differ between groups (Table 1; Figure 2A,B). No significant difference in sex ratio was observed between groups.

Regarding genetic features, patients with autoimmune disease had significantly larger D4Z4 repeat arrays compared with non‐autoimmune patients (7.18 ± 1.82 vs. 6.53 ± 1.81 repeat units, *p* = 0.01). Distribution analysis further demonstrated a marked shift toward larger repeat sizes in autoimmune patients. In the non‐autoimmune group, patients were similarly distributed between the 4–6 RU category (44.7%) and the 7–10 RU category (49.8%). In contrast, autoimmune patients were predominantly distributed in the 7–10 RU group (74.4%, *p* = 0.0001), whereas only 22.0% carried 4–6 RU alleles (*p* = 0.0003; Table 1; Figure 2C,D).

The distribution of CCEF categories differed significantly between groups. Specifically, while most of the non‐autoimmune patients were classified in the A category (*N* = 159, 73.27%), this proportion appeared significantly reduced in the autoimmune patients (*N* = 45, 54.88%, *p* = 0.003, Table 1 and Figure 2E). In contrast, autoimmune patients presented a significantly higher proportion of atypical patients, classified as D, compared to the non‐autoimmune patients (*N* = 25, 30.49% and *N* = 18, 8.29% respectively, *p* < 0.0001, Table 1 and Figure 2E). No difference was found in the proportion of patients classified as B or C in the 2 groups, nor in the proportions of the subcategories within each category (Figure 1A).

Importantly, patients with autoimmune disease exhibited significantly higher FSHD severity scores compared to those without autoimmune disease (7.98 ± 3.65 vs. 6.56 ± 3.51, *p* = 0.003, Table 1; Figure 2F).

### 3.4 Stratified Analyses According to CCEF Categories

**FIGURE 3:**
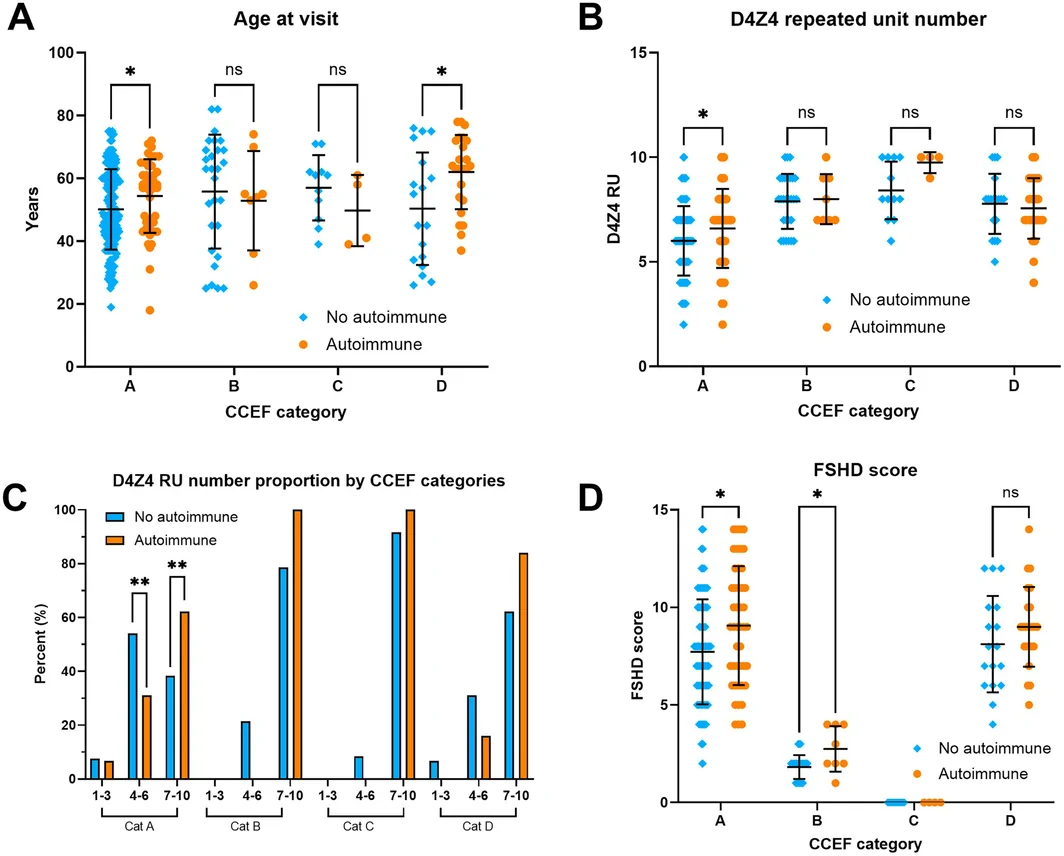
Stratified demographic, genetic, and clinical analyses according to CCEF category and autoimmune disease status. (A) Age at examination according to autoimmune status within each CCEF category. (B) D4Z4 repeat unit number according to autoimmune status within each CCEF category. (C) Distribution of D4Z4 RU categories within each CCEF category. (D) FSHD severity score according to autoimmune status within each CCEF category. Data are presented as mean ± SD. *p < 0.05, **p < 0.01.

Within CCEF categories, autoimmune patients in categories A and D were significantly older at examination compared to non‐autoimmune patients (category A: 54.38 ± 11.71 years and 50.11 ± 12.77 years, respectively, *p* = 0.03, and category D: 62 ± 11.83 years and 50.33 ± 17.89 years, respectively, *p* = 0.02), whereas no differences were observed in categories B and C. No differences were found in age at onset or disease duration across categories (Table S2; Figure 3A).

In category A, autoimmune patients had higher D4Z4 RU numbers compared to non‐autoimmune patients (6.6 ± 1.89 vs. 6.01 ± 1.66, *p* = 0.04), whereas no differences were observed in other categories (Table S2; Figure 3B). Consistent with the overall analysis, autoimmune patients in category A showed a shift toward higher RU categories, with a reduced proportion of patients with 4–6 D4Z4 RUs (*N* = 14, 31.11%, *p* = 0.007) and a higher proportion of patients in the range of 7–10 D4Z4 RUs (*N* = 28, 62.22%, *p* = 0.006) compared to non‐autoimmune patients (*N* = 86, 54.09% and *N* = 61, 38.36%, respectively, Table S2; Figure 3C).

Regarding clinical severity, autoimmune patients in categories A and B had significantly higher FSHD scores compared to their non‐autoimmune counterparts (9.07 ± 3.05 vs. 7.72 ± 2.69 for category A and 2.75 ± 1.17 vs. 1.82 ± 0.61 for category B, both *p* = 0.004) (Table S2; Figure 3D). Results from categories with very small sample sizes should be interpreted cautiously. Overall, these findings indicate a consistent association between autoimmune disease, higher D4Z4 repeat unit numbers, and increased disease severity.

### 3.5 Multivariable Analysis of Factors Associated With FSHD Severity

To further investigate the independent and combined effects of autoimmune disease and D4Z4 repeat unit number, on FSHD severity, we performed a multivariable linear regression analysis (Table 3 and Figure S1). Higher D4Z4 repeat unit numbers were significantly associated with lower disease severity (β = −0.96, 95% CI [−1.14 to −0.78], *p* < 0.0001), whereas longer disease duration was associated with increased severity (β = 0.11, 95% CI [0.08 to 0.14], *p* < 0.0001). Age showed a small but significant negative association with severity (β = −0.02, 95% CI [−0.05 to −0.002], *p* = 0.03), while sex was not significantly associated with disease severity (*p* = 0.67; Table 3 and Figure S1).

**TABLE 3:** Multivariable linear regression analysis of factors associated with FSHD severity.

Parameter estimates	Variable	Estimate	95% CI (profile likelihood)	|t|	*p*	*p* value summary
β0	Intercept	12.17	10.70 to 13.63	16.30	< 0.0001	****
β1	Age	−0.02516	−0.04839 to −0.001934	2.132	0.0338	*
β2	Sex[F]	0.1239	−0.4466 to 0.6943	0.4273	0.6695	ns
β3	D4Z4 RU	−0.9548	−1.138 to −0.7711	10.23	< 0.0001	****
β4	Disease duration	0.1087	0.08148 to 0.1360	7.850	< 0.0001	****
β5	Autoimmune[Y]	2.115	1.472 to 2.759	6.471	< 0.0001	****

*Note:* Results are presented as regression coefficients (β) with 95% confidence intervals (CI). The multivariable linear regression model included age, sex, D4Z4 RU number, disease duration, and autoimmune disease status. Male and absence of autoimmune disease was used as the reference group. Positive β coefficients indicate increased FSHD severity scores, whereas negative β coefficients indicate lower severity scores. Statistical significance was assessed using two‐sided tests, and statistical significance was defined as *p < 0.05, **p < 0.01, ***p < 0.001, ****p < 0.0001.

Importantly, the presence of autoimmune disease remained independently associated with increased FSHD severity after adjustment for all covariates (β = 2.12, 95% CI [1.47 to 2.76], *p* < 0.0001), indicating that patients with autoimmune disease have, on average, higher severity scores after adjustment for all covariates (Table 3 and Figure S1).

**FIGURE 4:**
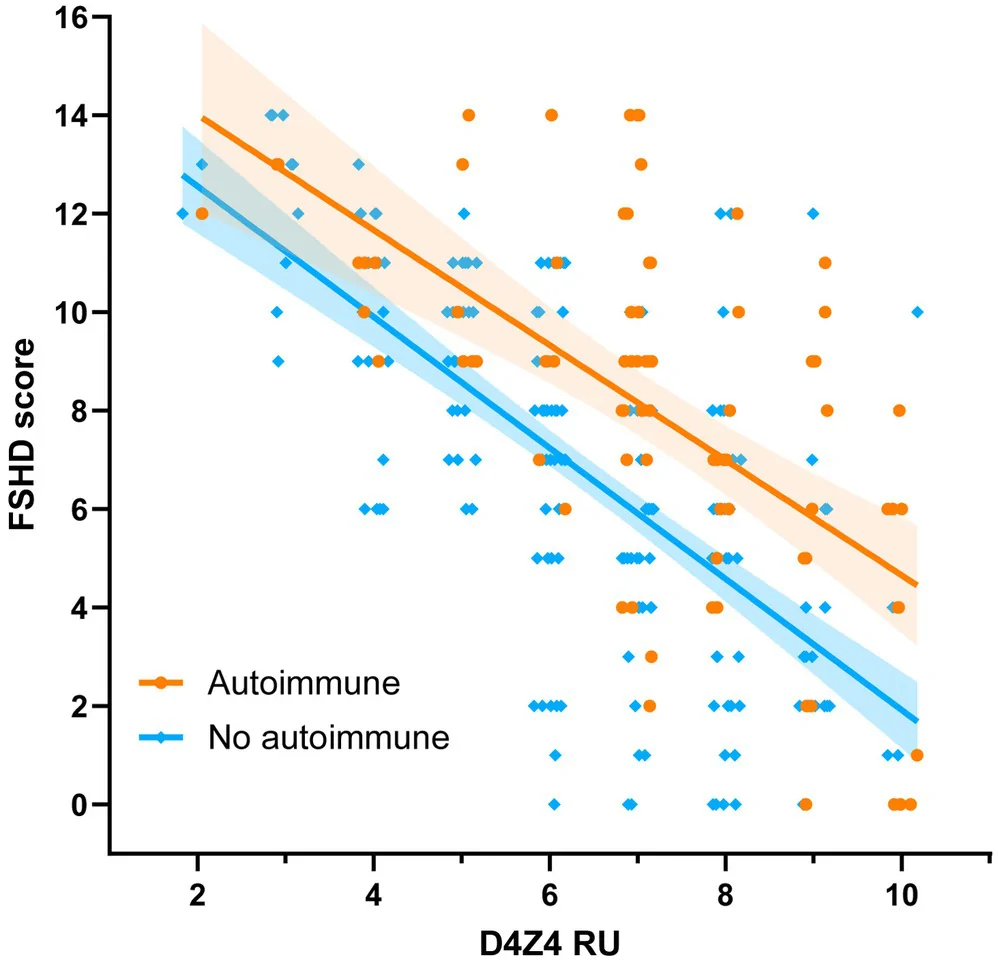
Relationship between D4Z4 repeat unit number and FSHD severity according to autoimmune disease status. Scatter plot showing FSHD severity score as a function of D4Z4 repeat unit (RU) number in FSHD1 patients with and without autoimmune disease. A small horizontal jitter was applied to the data points to reduce overlap and improve visualization. Linear regression lines and 95% confidence intervals are shown for each group.

Consistent with these findings, graphical analysis showed a negative relationship between D4Z4 repeat unit number and FSHD severity in both groups (Figure 4). Patients with autoimmune disease exhibited consistently higher severity scores across the range of repeat unit values, with parallel regression lines indicating no significant interaction between autoimmune status and D4Z4 repeat units.

## 4 Discussion

In this study, we demonstrate that autoimmune diseases are frequent in patients with FSHD1 and are associated with increased disease severity independently of major demographic, genetic, and clinical determinants. Although patients with autoimmune comorbidities carried significantly larger D4Z4 repeat arrays, which are generally associated with milder disease phenotypes [20], they nevertheless exhibited higher FSHD severity scores compared with patients without autoimmune disease. Importantly, multivariable analyses confirmed that autoimmune disease remained independently associated with increased disease severity after adjustment for age, sex, disease duration, D4Z4 RU number, and CCEF category. Together, these findings support the hypothesis that autoimmune conditions may act as independent modifiers of disease severity in FSHD.

The inverse relationship between D4Z4 repeat size and disease severity is well established in FSHD1. Several cohort studies have shown that shorter D4Z4 arrays are associated with earlier onset and more severe muscle involvement, although substantial interindividual variability remains unexplained [8, 22]. In our cohort, however, autoimmune patients carried significantly larger D4Z4 repeat arrays despite exhibiting greater clinical severity. Moreover, we did not identify a significant interaction between genetic burden and autoimmune status, and regression analyses showed similar relationships between D4Z4 RU number and clinical severity in patients with and without autoimmune diseases. These findings suggest that autoimmune comorbidities may influence disease severity by acting as a potential disease modifier through mechanisms at least partially independent of the primary genetic defect.

The spectrum of autoimmune diseases identified in our cohort was broad and included endocrine, dermatological, hepatological, neurological, and systemic autoimmune disorders. Several conditions appeared overrepresented compared with published population estimates, particularly autoimmune type I diabetes, myasthenia gravis, primary biliary cholangitis, inflammatory myopathies, and Sjögren's syndrome. Reported prevalences in the European general population are approximately 0.125% for autoimmune diabetes [39], 0.02% for myasthenia gravis [40], 0.022% for primary biliary cholangitis [41], and approximately 0.061% for Sjögren's syndrome [42]. Although comparisons with published population prevalence estimates should be interpreted cautiously because of methodological heterogeneity and the small number of cases for some disorders, our findings may also have been influenced by the referral setting of our cohort. As a national neuromuscular referral center, our cohort may include patients with more complex clinical phenotypes who undergo more comprehensive medical evaluations, potentially increasing the detection and documentation of autoimmune comorbidities. Conversely, common and generally mild autoimmune conditions, such as atopic dermatitis, may remain underreported if they are not systematically documented in neurological medical records. Nevertheless, the enrichment observed for several autoimmune neuromuscular and systemic diseases is noteworthy. In contrast, common inflammatory skin disorders such as psoriasis and atopic dermatitis did not appear overrepresented, suggesting that the association may involve specific autoimmune mechanisms rather than generalized inflammatory susceptibility.

These observations are consistent with accumulating evidence suggesting a broader interplay between immune dysregulation and FSHD pathophysiology. Several case reports and small series have previously described the coexistence of FSHD and myasthenia gravis or idiopathic inflammatory myositis, raising the possibility of shared or interacting immune‐mediated mechanisms between the two disorders [29, 30]. A recent study further highlighted the potential contribution of inflammatory mechanisms in FSHD, reporting associations between serum interleukin‐6 (IL‐6) levels and clinical disease expression [17]. Together with our findings and prior reports linking FSHD with autoimmune neuromuscular disease, these data support the emerging concept that immune‐mediated pathways may represent important modifiers of disease penetrance, severity, and phenotypic variability in FSHD.

The mechanisms linking autoimmunity and FSHD severity remain incompletely understood. FSHD is increasingly recognized as a complex multisystem disorder involving epigenetic dysregulation and aberrant expression of the *DUX4* transcription factor [43, 44]. *DUX4* expression induces oxidative stress [12], immune activation [16, 17, 23], and muscle toxicity through activation of inflammatory and innate immune pathways [15]. Inflammatory infiltrates have also been described in muscle biopsies from FSHD patients, supporting a role for immune dysregulation in disease progression [23, 45]. Chronic muscle injury may promote immune activation through release of intracellular antigens and pro‐inflammatory mediators [31, 32], whereas autoimmune diseases may further exacerbate muscle dysfunction through systemic inflammation, immune‐mediated tissue injury, or impaired regenerative responses [46].

Interestingly, autoimmune patients were more frequently classified in CCEF category D, corresponding to atypical phenotypes [37], and showed higher FSHD severity scores within categories A and B. These findings raise the possibility that autoimmune comorbidities may contribute not only to disease severity but also to phenotypic heterogeneity in FSHD. On the other hand, no differences were found in disease severity when comparing autoimmune and non‐autoimmune patients classified as category D. We have already described the population composed of category D patients, which was mostly made up of patients with another concomitant unrelated genetic disease [38]. We found that most of these patients did not display an autoimmune condition. While autoimmune diseases may act as disease modifiers for typical patients, leading to a more severe disease presentation, for atypical patients, disease severity seems to be related to the presence of the second concomitant condition responsible for the atypical features, whether of acquired or genetic origin. Whether these atypical presentations reflect shared pathogenic mechanisms, increased diagnostic complexity, or cumulative systemic burden remains unclear.

Notably, autoimmune disease was substantially more frequent among patients carrying 7–10 D4Z4 repeat units than among those carrying shorter alleles (36.1% vs. 16.2%). Consistent with this enrichment, 73 of the 96 autoimmune conditions identified in the cohort occurred in patients belonging to the 7–10 RU subgroup. These findings support an unexpected association between larger residual D4Z4 repeat arrays and autoimmune manifestations in FSHD1. The observation that autoimmune patients carried larger D4Z4 repeat arrays despite exhibiting greater disease severity suggests that factors other than the primary genetic burden contribute to disease expression in this subgroup.

These findings resonate with our recent description of “double trouble” cases, in which FSHD is associated with a second, unrelated genetic disorder. In that study, most FSHD1 patients carrying an unrelated genetic condition, as well as patients presenting uncommon clinical features, harbored disease alleles in the 7–10 D4Z4 RU range [38]. Taken together, these findings raise the possibility that the 7–10 D4Z4 RU range defines a subgroup of FSHD1 patients in whom disease expression is particularly susceptible to secondary modifiers. In these individuals, autoimmune diseases, unrelated genetic disorders, or other yet unidentified factors may contribute disproportionately to phenotypic variability and clinical severity, helping to explain part of the heterogeneity that remains unexplained by D4Z4 repeat size alone.

**FIGURE 5:**
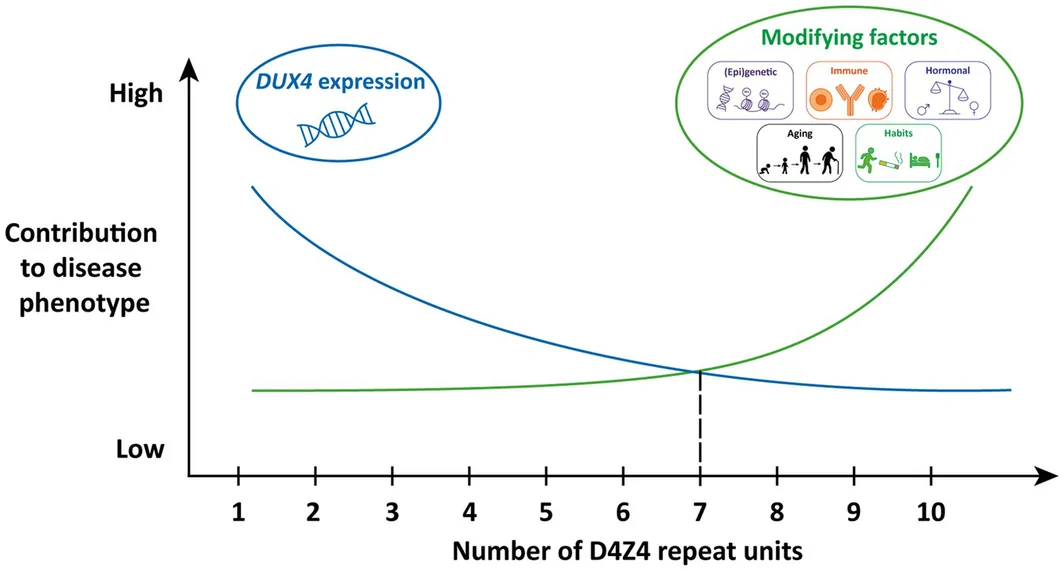
Proposed model of disease modifiers across the D4Z4 repeat size spectrum in FSHD1.

One possible explanation is that individuals with larger repeat arrays may require additional nongenetic modifiers, including inflammatory or autoimmune mechanisms, to develop clinically significant disease manifestations (Figure 5). Interestingly, autoimmune patients displayed a significantly later disease onset than patients without autoimmune disease. This supports the hypothesis that an altered immune background, along with aging and immunosenescence, which contributes to a pro‐inflammatory state, may influence disease severity and presentation, especially in patients with larger alleles. Although our data do not establish causality, they support the hypothesis that the clinical consequences of D4Z4 repeat size may extend beyond muscle involvement and influence susceptibility to additional modifiers contributing to disease heterogeneity.

Considering the existing literature [30, 38] and the present findings, we propose a conceptual model in which the relative contribution of disease modifiers may vary according to residual D4Z4 repeat size. In patients carrying shorter alleles (< 7 RU), disease severity and clinical expression appear to be driven predominantly by the primary D4Z4 contraction. In contrast, in patients carrying larger residual alleles (≥ 7 RU), additional modifying factors may play a proportionally greater role in shaping disease phenotype and severity (Figure 5). Several such modifiers have already been implicated in FSHD, including genetic and epigenetic factors [38, 47], hormones [48], telomere shortening [49], environmental and lifestyle factors [50], aging [5] and, based on the present study, autoimmune diseases. These factors may act individually or synergistically, contributing to both clinical severity and phenotypic heterogeneity. Such a model could help explain why patients with similar D4Z4 repeat sizes, particularly within the 7–10 RU range, may exhibit markedly different clinical presentations and disease trajectories.

Several limitations should be acknowledged. First, this was a retrospective single‐center study, potentially introducing selection and referral biases. Second, autoimmune diseases were heterogeneous and analyzed collectively despite potentially distinct pathogenic mechanisms. Third, comparisons with general population prevalence estimates relied on epidemiological studies derived from different populations and methodologies. Finally, several autoimmune diseases were represented by small numbers of patients, resulting in limited statistical power and wide confidence intervals for rare conditions.

Despite these limitations, our study provides evidence supporting an association between autoimmune diseases and increased clinical severity in FSHD1. These findings highlight the importance of systematically assessing autoimmune comorbidities in FSHD patients and suggest that immune‐related mechanisms may contribute to disease modulation. Further multicenter and longitudinal studies will be required to clarify the biological basis of these associations and determine whether immune‐targeted therapeutic strategies could influence disease progression in selected patients.

## Author Contributions

J.P. and S.S. contributed to the study's conception and design. G.T., A.E., M.G., B.S., L.V., M.C., M.‐B.I., A.P., and S.S. contributed to the data acquisition. J.P. and S.S. contributed to data analysis. J.P. wrote the manuscript and prepared the Figures. G.T. and S.S. contributed to the manuscript preparation.

## Funding

The authors have nothing to report.

## Conflicts of Interest

The authors declare no conflicts of interest.

## Supporting information


**Table S1:** Published prevalence estimates of autoimmune diseases used for observed‐to‐expected ratio calculations. Published prevalence estimates from the general population used to calculate expected case numbers and observed‐to‐expected ratios in the FSHD1 cohort. Prevalence values were obtained from epidemiological studies, systematic reviews, meta‐analyses, or international disease registries.
**Table S2:** Demographic, genetic, and clinical characteristics according to CCEF category and autoimmune disease status. Continuous variables are presented as mean ± standard deviation (SD), and categorical variables as number and percentage. Comparisons between patients with and without autoimmune disease were performed within each CCEF category using unpaired t‐tests for continuous variables and Fisher's exact test for categorical variables. D4Z4 RU categories were defined as 1–3, 4–6, and 7–10 repeat units. Clinical severity was classified according to the CCEF: category A, classical phenotype; category B, incomplete phenotype; category C, asymptomatic or pauci‐symptomatic phenotype; and category D, atypical phenotype. Statistical significance was defined as p < 0.05. NS: nonsignificant.
**Figure S1:** Forest plot of factors associated with FSHD severity in multivariable analysis. Regression coefficients (β) and corresponding 95% confidence intervals from the multivariable linear regression model assessing associations with FSHD severity score. The model included age, sex, D4Z4 repeat unit number, disease duration, and autoimmune disease status. Male and absence of autoimmune disease served as reference groups.

## Data Availability

The data that support the findings of this study are available from the corresponding author on reasonable request.
