# Supplementary material for: Autoimmune Comorbidities as Modifiers of Phenotypic Heterogeneity in Facioscapulohumeral Dystrophy
Source: Ann Clin Transl Neurol. 2026 Sep 22:10.1002/acn3.70529. Online ahead of print. doi: 10.1002/acn3.70529 (PMC13595050; doi:10.1002/acn3.70529)
Supplement: Supplementary file 1 [file ACN3-9999-0-s001.docx]

**Supplementary table 1: Published prevalence estimates of autoimmune diseases used for observed-to-expected ratio calculations**

| Autoimmune disease | General population prevalence (%) | Methods | Reference |
| --- | --- | --- | --- |
| Hashimoto’s Thyroiditis | 7.8 | Systematic review / meta-analysis | Hu et al., 2022 |
| Autoimmune Diabetes | 0.13 | Systematic review / meta-analysis | Mobasseri et al., 2024, 2020 |
| Atopic Dermatitis | 17.1 | Systematic review | Bylund et al., 2020 |
| Myasthenia gravis | 0.02 | Systematic review | Sciancalepore et al., 2024 |
| Primary Biliary Cholangitis | 0.022 | Systematic review / meta-analysis | Gazda et al., 2021 |
| Psoriasis | 1.83 | Systematic review / meta-analysis | Parisi et al., 2020 |
| Inflammatory Myositis | 0.014 | Systematic review | Meyer et al., 2015 |
| Sjögren’s Syndrome | 0.061 | Systematic review / meta-analysis | Qin et al., 2015 |
| Addison’s Disease | 0.01 | Review / epidemiological prevalence | Betterle et al., 2019 |
| Vitiligo | 0.2 | Retrospective study | Estebaranz et al., 2024 |
| Alopecia | 2 | Systematic review / meta-analysis | Lee et al., 2020 |
| Multiple Sclerosis | 0.133 | Epidemiological study | Walton et al., 2020 |
| CIDP | 0.0028 | Meta-analysis | Broers et al., 2019 |
| Rheumatoid Arthritis | 0.35 | Review / epidemiological prevalence | Uke et al., 2025 |

Published prevalence estimates from the general population used to calculate expected case numbers and observed-to-expected ratios in the FSHD1 cohort. Prevalence values were obtained from epidemiological studies, systematic reviews, meta-analyses, or international disease registries.

**REFERENCES**

Betterle, C., Presotto, F., Furmaniak, J., 2019. Epidemiology, pathogenesis, and diagnosis of Addison’s disease in adults. J Endocrinol Invest 42, 1407–1433. https://doi.org/10.1007/s40618-019-01079-6

Broers, M.C., Bunschoten, C., Nieboer, D., Lingsma, H.F., Jacobs, B.C., 2019. Incidence and Prevalence of Chronic Inflammatory Demyelinating Polyradiculoneuropathy: A Systematic Review and Meta-Analysis. Neuroepidemiology 52, 161–172. https://doi.org/10.1159/000494291

Bylund, S., Kobyletzki, L.B., Svalstedt, M., Svensson, Å., 2020. Prevalence and Incidence of Atopic Dermatitis: A Systematic Review. Acta Derm Venereol 100, adv00160. https://doi.org/10.2340/00015555-3510

Estebaranz, J.L.L., González-Montagut, C.M., González, J., Miguel, I.A.G.S., Garcia-Recio, V., Callejo, D., De Prado, A., Delattre, C., 2024. Real-world evidence for vitiligo using an electronic medical records database in Spain: the REVEAL-ES study. Eur J Dermatol 34, 251–259. https://doi.org/10.1684/ejd.2024.4676

Gazda, J., Drazilova, S., Janicko, M., Jarcuska, P., 2021. The Epidemiology of Primary Biliary Cholangitis in European Countries: A Systematic Review and Meta-Analysis. Can J Gastroenterol Hepatol 2021, 9151525. https://doi.org/10.1155/2021/9151525

Hu, X., Chen, Y., Shen, Y., Tian, R., Sheng, Y., Que, H., 2022. Global prevalence and epidemiological trends of Hashimoto’s thyroiditis in adults: A systematic review and meta-analysis. Front Public Health 10, 1020709. https://doi.org/10.3389/fpubh.2022.1020709

Lee, H.H., Gwillim, E., Patel, K.R., Hua, T., Rastogi, S., Ibler, E., Silverberg, J.I., 2020. Epidemiology of alopecia areata, ophiasis, totalis, and universalis: A systematic review and meta-analysis. Journal of the American Academy of Dermatology 82, 675–682. https://doi.org/10.1016/j.jaad.2019.08.032

Meyer, A., Meyer, N., Schaeffer, M., Gottenberg, J.-E., Geny, B., Sibilia, J., 2015. Incidence and prevalence of inflammatory myopathies: a systematic review. Rheumatology (Oxford) 54, 50–63. https://doi.org/10.1093/rheumatology/keu289

Mobasseri, M., Shirmohammadi, M., Amiri, T., Vahed, N., Hosseini Fard, H., Ghojazadeh, M., 2024. Corrigendum to: Prevalence and incidence of type 1 diabetes in the world: a systematic review and meta-analysis Mobasseri M, Shirmohammadi M, Amiri T, Vahed N, Hosseini Fard H, Ghojazadeh M. Health Promot Perspect. 2020 Mar 30;10(2):98-115. doi: 10.34172/hpp.2020.18. Health Promot Perspect 14, 202–205. https://doi.org/10.34172/hpp.43143

Mobasseri, M., Shirmohammadi, M., Amiri, T., Vahed, N., Hosseini Fard, H., Ghojazadeh, M., 2020. Prevalence and incidence of type 1 diabetes in the world: a systematic review and meta-analysis. Health Promot Perspect 10, 98–115. https://doi.org/10.34172/hpp.2020.18

Parisi, R., Iskandar, I.Y.K., Kontopantelis, E., Augustin, M., Griffiths, C.E.M., Ashcroft, D.M., 2020. National, regional, and worldwide epidemiology of psoriasis: systematic analysis and modelling study. BMJ m1590. https://doi.org/10.1136/bmj.m1590

Qin, B., Wang, J., Yang, Z., Yang, M., Ma, N., Huang, F., Zhong, R., 2015. Epidemiology of primary Sjögren’s syndrome: a systematic review and meta-analysis. Ann Rheum Dis 74, 1983–1989. https://doi.org/10.1136/annrheumdis-2014-205375

Sciancalepore, F., Lombardi, N., Valdiserra, G., Bonaso, M., Cappello, E., Hyeraci, G., Crescioli, G., Celani, M.G., Cantisani, T.A., Brunori, P., Vecchi, S., Bacigalupo, I., Locuratolo, N., Lacorte, E., Vanacore, N., Kirchmayer, U., 2024. Prevalence, Incidence, and Mortality of Myasthenia Gravis and Myasthenic Syndromes: A Systematic Review. Neuroepidemiology 59, 579–592. https://doi.org/10.1159/000539577

Uke, P., Maharaj, A., Adebajo, A., 2025. A review on the epidemiology of rheumatoid arthritis: An update and trends from current literature. Best Pract Res Clin Rheumatol 39, 102036. https://doi.org/10.1016/j.berh.2025.102036

Walton, C., King, R., Rechtman, L., Kaye, W., Leray, E., Marrie, R.A., Robertson, N., La Rocca, N., Uitdehaag, B., Van Der Mei, I., Wallin, M., Helme, A., Angood Napier, C., Rijke, N., Baneke, P., 2020. Rising prevalence of multiple sclerosis worldwide: Insights from the Atlas of MS, third edition. Mult Scler 26, 1816–1821. https://doi.org/10.1177/1352458520970841

**Supplementary table 2: Demographic, genetic, and clinical characteristics according to CCEF category and autoimmune disease status.**

|  |  | No autoimmune disease | | | | Autoimmune disease | | | | p value | | | |
| --- | --- | --- | --- | --- | --- | --- | --- | --- | --- | --- | --- | --- | --- |
|  | CCEF category | A | B | C | D | A | B | C | D | A | B | C | D |
|  | N  (%) | 159  (73.27) | 28  (12.9) | 12  (5.53) | 18  (8.29) | 45  (54.88) | 8  (9.76) | 4  (4.88) | 25  (30.49) |  |  |  |  |
| Age  (years) | Mean  (SD) | 50.11 (12.77) | 55.79 (18.14) | 57  (10.41) | 50.33 (17.89) | 54.38 (11.71) | 52.88 (15.84) | 49.75 (11.35) | 62  (11.83) | p = 0.03  * | NS | NS | p = 0.02  * |
| Age at onset (years) | Mean  (SD) | 31.45 (13.9) | 42.61 (20.24) | 56.29 (8.48) | 31  (16.14) | 33.53 (13.91) | 38  (21.19) | 53  (0) | 47.13  (18) | NS | NS | NS | NS |
| Disease duration (years) | Mean  (SD) | 18.67 (10.1) | 13.18 (16.86) | 2.17 (2.66) | 19.33 (9.95) | 20.84 (12.22) | 14.88 (12.55) | 1.25  (2.5) | 14.88 (11.76) | NS | NS | NS | NS |
| D4Z4 Repeat Unit | Mean  (SD) | 6.01 (1.66) | 7.89 (1.32) | 8.42 (1.38) | 7.78 (1.44) | 6.6  (1.89) | 8  (1.19) | 9.75  (0.5) | 7.56 (1.45) | p = 0.04  * | NS | p = 0.08 Trends | NS |
|  | 1-3  (%) | 12  (7.55) | 0  (0) | 0  (0) | 0  (0) | 3  (6.67) | 0  (0) | 0  (0) | 0  (0) | NS | NS | NS | NS |
|  | 4-6  (%) | 86  (54.09) | 6  (21.43) | 1  (8.33) | 4  (22.22) | 14  (31.11) | 0  (0) | 0  (0) | 4  (16) | p = 0.007 ** | NS | NS | NS |
|  | 7-10  (%) | 61  (38.36) | 22  (78.57) | 11  (91.67) | 14  (77.78) | 28  (62.22) | 8  (100) | 4  (100) | 21  (84) | p = 0.006 ** | NS | NS | NS |
| FSHD Score | Mean  (SD) | 7.72 (2.69) | 1.82 (0.61) | 0  (0) | 8.12 (2.47) | 9.07 (3.05) | 2.75 (1.17) | 0  (0) | 9  (2.04) | p = 0.004 ** | p = 0.004 ** | NS | NS |

Continuous variables are presented as mean ± standard deviation (SD), and categorical variables as number and percentage. Comparisons between patients with and without autoimmune disease were performed within each CCEF category using unpaired t-tests for continuous variables and Fisher’s exact test for categorical variables. D4Z4 RU categories were defined as 1–3, 4–6, and 7–10 repeat units. Clinical severity was classified according to the CCEF: category A, classical phenotype; category B, incomplete phenotype; category C, asymptomatic or pauci-symptomatic phenotype; and category D, atypical phenotype. Statistical significance was defined as p < 0.05. NS: non-significant.

**Supplementary Figure 1. Forest plot of factors associated with FSHD severity in multivariable analysis.**

Regression coefficients (β) and corresponding 95% confidence intervals from the multivariable linear regression model assessing associations with FSHD severity score. The model included age, sex, D4Z4 repeat unit number, disease duration, and autoimmune disease status. Male and absence of autoimmune disease served as reference groups.
